# Supplementary material for: The interactome of intact mitochondria by cross-linking mass spectrometry provides evidence for coexisting respiratory supercomplexes
Source: Mol Cell Proteomics. 2017 Dec 8;17(2):216–32. doi: 10.1074/mcp.RA117.000470 (PMC5795388; doi:10.1074/mcp.RA117.000470)
Supplement: Supplemental Data [file supp_RA117.000470_133922_0_supp_23978_fzffwf.zip › spectra_annotation/mito_DR_spectra_annotation/411-1-5-1-3-1.pdf]

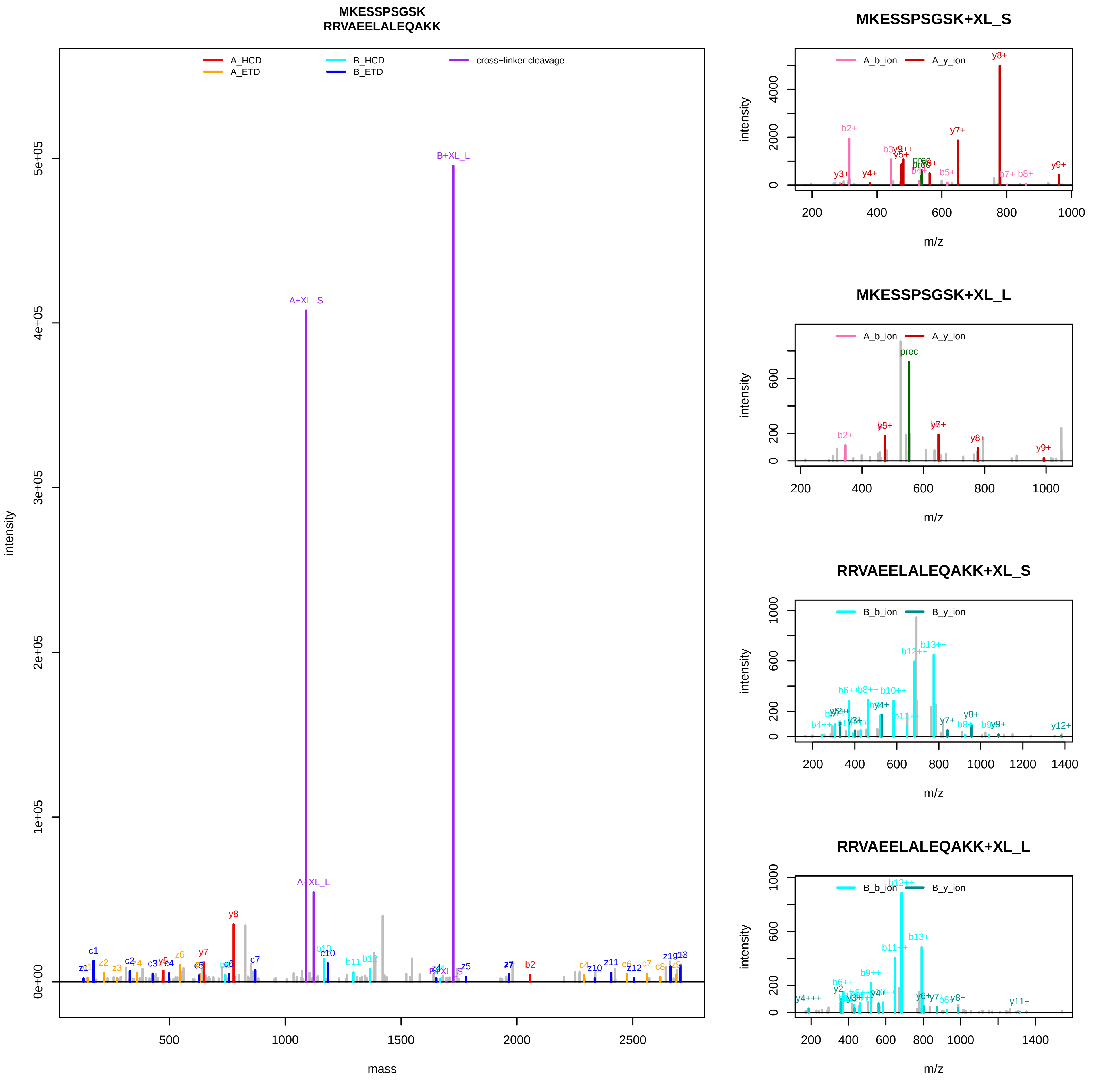

MKESSPSGSK
RRVAEELALEQAKK
A_HCD
A_ETD
B_HCD
B_ETD
cross−linker cleavage
B+XL_L
A+XL_S
A+XL_L
y8
y7
y5
c1
c2
c3
c4
c5
c6
c7
c10
b10
b11
b12
z2
z3
z4
z5
z6
z7
z10
z11
z12
z9
c4
c6
c7
c8
b2
intensity
mass
MKESSPSGSK+XL_S
A_b_ion
A_y_ion
y8+
b2+
y7+
y9+
y3+
y4+
y5+
b5+
b7+
b8+
prec
m/z
intensity
MKESSPSGSK+XL_L
A_b_ion
A_y_ion
prec
b2+
y5+
y7+
y8+
y9+
m/z
intensity
RRVAEELALEQAKK+XL_S
B_b_ion
B_y_ion
b12++
b13++
b6++
b8++
b10++
b11++
b4++
y4+
y7+
y8+
y9+
y12+
m/z
intensity
RRVAEELALEQAKK+XL_L
B_b_ion
B_y_ion
b12++
b13++
b11++
b9++
b6++
y4+++
y2+
y6+
y7+
y8+
y11+
m/z
intensity
